# General Anesthetics Predicted to Block the GLIC Pore with Micromolar Affinity

**DOI:** 10.1371/journal.pcbi.1002532

**Published:** 2012-05-31

**Authors:** David N. LeBard, Jérôme Hénin, Roderic G. Eckenhoff, Michael L. Klein, Grace Brannigan

**Affiliations:** 1 Department of Chemistry and Institute for Computational Molecular Science, Temple University, Philadelphia, Pennsylvania, United States of America; 2 Laboratoire d'Ingénierie des Structures Macromoléculaires, CNRS and Aix-Marseille University, Marseille, France; 3 Department of Anesthesiology and Critical Care, University of Pennsylvania School of Medicine, Philadelphia, Pennsylvania, United States of America; 4 Department of Physics and Center for Computational and Integrative Biology, Rutgers University-Camden, Camden, New Jersey, United States of America; NASA Ames Research Center, United States of America

## Abstract

Although general anesthetics are known to modulate the activity of ligand-gated ion channels in the Cys-loop superfamily, there is at present neither consensus on the underlying mechanisms, nor predictive models of this modulation. Viable models need to offer quantitative assessment of the relative importance of several identified anesthetic binding sites. However, to date, precise affinity data for individual sites has been challenging to obtain by biophysical means. Here, the likely role of pore block inhibition by the general anesthetics isoflurane and propofol of the prokaryotic pentameric channel GLIC is investigated by molecular simulations. Microscopic affinities are calculated for both single and double occupancy binding of isoflurane and propofol to the GLIC pore. Computations are carried out for an open-pore conformation in which the pore is restrained to crystallographic radius, and a closed-pore conformation that results from unrestrained molecular dynamics equilibration of the structure. The GLIC pore is predicted to be blocked at the micromolar concentrations for which inhibition by isofluorane and propofol is observed experimentally. Calculated affinities suggest that pore block by propofol occurs at signifcantly lower concentrations than those for which inhibition is observed: we argue that this discrepancy may result from binding of propofol to an allosteric site recently identified by X-ray crystallography, which may cause a competing gain-of-function effect. Affinities of isoflurane and propofol to the allosteric site are also calculated, and shown to be 3 mM for isoflurane and 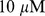 for propofol; both anesthetics have a lower affinity for the allosteric site than for the unoccupied pore.

## Author Summary

Although general anesthesia is performed every day on thousands of people, its detailed microscopic mechanisms are not known. What is known is that general anesthetic drugs modulate the activity of ion channels in the central nervous system. These channels are proteins that open in response to binding of neurotransmitter molecules, creating an electric current through the cell membrane and thus propagating nerve impulses between cells. One possible mechanism for ion channel inhibition by anesthetics is that the drugs bind inside the pore of the channels, blocking ion current. Here we investigate such a pore block mechanism by computing the strength of the drugs' interaction with the pore – and hence the likelihood of binding, in the case of GLIC, a bacterial channel protein. The results, obtained from numerical simulations of atomic models of GLIC, indicate that the anesthetics isoflurane and propofol have a tendency to bind in the pore that is strong enough to explain blocking of the channel, even at low concentration of the drugs.

## Introduction

Despite more than a century of research [1], [2], open questions remain regarding the molecular mechanism by which anesthetics modulate signal transmissions in the central nervous system (CNS). Electrophysiology and photolabeling have demonstrated that general anesthetics bind to the Cys-loop superfamily of pentameric ligand-gated ion channels. [3]–[5]. Several high resolution crystal structures have helped decipher the binding modes of anesthetics to proteins [6]–[9]. Only recently, however, did crystal structures [10] of the general anesthetics desflurane and propofol in complex with a prokaryotic member of the superfamily (GLIC) appear, providing an atomic-level basis to the “direct binding” hypothesis for modulation of Cys-loop receptors by general anesthetics. Several open questions cannot be addressed by crystallography alone, however, including energetics of binding, the possible role of pore block (due to detergents lodged in the pore during crystallization), and the molecular mechanism by which binding to allosteric sites modulates function. Potential differences in binding modes between prokaryotic and eukaryotic channels further complicates interpretation of results. Consequently, a full understanding of the physical mechanism through which binding of anesthetics to Cys-loop ion channels affects ion permeation remains elusive, despite numerous experimental [4], [10]–[19] and computational [20]–[26] studies.

Ligand-gated ion channels in the Cys-loop receptor superfamily are sensitive to general anesthetics at clinical concentrations [4], [14]; in general, excitatory cationic channels are inhibited by general anesthetics, while inhibitory anionic channels are potentiated. Members of this superfamily include the anion permeable glycine and 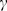-aminobutyric acid type A (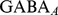) receptors, as well as the cation permeable serotonin (5-HT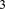) and nicotinic acetylcholine (nAChR) receptors. A cation channel in the Cys-loop family from the bacteria *Gloeobacter violaceus* (GLIC) has been crystallized at atomic resolution (2.9 and 3.1 Å) in a putatively open state [27], [28]. While gated by protons rather than neurotransmitters, GLIC retains a large amount of structural similarity with Cys-loop receptors including the pentameric symmetry, an extracellular domain (ECD) with a predominantly beta structure, and four transmembrane alpha helices (M1–M4) per subunit. Patch clamp experiments revealed that a diverse group of molecules, including both injected and inhaled anesthetics, inhibit GLIC at subclinical concentrations [10], [29]. An additional structure for the Glutamate-gated anion channel from *C. Elegans* has been recently published [30], but the anesthetic sensitivity of this channel has not been reported.

Information regarding anesthetic binding sites within Cys-loop receptors has been obtained from molecular biology [11], [12], photoaffinity labelling [15]–[17], and NMR [13], [18], [19] experiments. X-ray structures of GLIC indicate an intra-subunit site for both volatile (desflurane) and injected (propofol) anesthetics in the outer part (extracellular side) of the transmembrane domain (TMD) [10]. No general anesthetic has been resolved in the transmembrane pore, but this may be precluded by binding of detergents (dodecylmaltoside, DDM) within the pore [27]. In one case, a brominated variant of the local anesthetic lidocaine has been detected inside the GLIC pore in a crystal structure by Hilf et al. [31] (PDB accession code 2XQ3). Only the large electron density of the bromine atom could be resolved, and its location indicates a binding mode for bromo-lidocaine that is compatible with binding of a bundle of DDM molecules (Supporting Figure S3). Results from electrophysiology [32], mutagenesis [11], and photoaffinity labeling [17] are consistent with occupation of the eukaryotic Cys-loop pore by anesthetics. Furthermore, in recent atomistic molecular dynamics (MD) simulations by our group [24], which identified several anesthetic binding sites for isoflurane in both nAChR and GLIC using long-time molecular dynamics for a flexible dock (“flooding”), two isoflurane molecules bound stably for 300 ns to the pores of both GLIC and nAChR. Similarly, a single isoflurane was observed to bind to the GLIC pore in Ref. [26]. Since there are significant expected ramifications of pore block for inhibition of ion channel function, it is difficult to discern the role of other allosteric sites without consideration of the potential for pore block at any concentrations at which inhibition is observed.

Due to the multitude [15], [22], [24], [25], [31], [33], [34] of binding sites in eukaryotic receptors indicated by both experiments and simulation, untangling the molecular mechanism of action requires a method for ranking detected sites according to their affinity and implications for channel function. Isolating individual sites remains unfeasible in experiments, and any measured affinities would reflect an average over multiple unknown sites. Computer simulations offer several techniques for calculating binding affinities for given anesthetics in individual binding pockets. One method, known as alchemical free energy perturbation (FEP), has been developed to obtain *absolute* binding free energies from MD simulations [35], [36]. FEP has already been used to calculate affinities for isoflurane binding to apoferritin that compare well to experiment [37], and used to characterize several potential binding sites for halothane on a neuronal nAChR [22]. Recent advances in computational techniques and resources have made the reliable calculation of binding affinities in systems as large as Cys-loop receptors feasible. Since FEP-MD simulations require high-resolution protein structures as input, GLIC serves as the most structurally relevant model for analyzing the energetics of anesthetic binding, and for probing a pore block event in detail.

In this article, we present an extensive quantitative FEP study of inhaled and injected anesthetics bound to the GLIC pore, considered primarily in the context of a pore-block mechanism. In over 1.5 microseconds of atomistic simulations, we calculate binding affinities for monomers and dimers of isoflurane and propofol for comparison with dose response curves from electrophysiology. The affinity of ethanol (which does not affect GLIC at mM concentrations) is also calculated as a negative control [29]. An analytical model for relating microscopic and macroscopic parameters of binding is presented and used to demonstrate that a pore-block model is sufficient to predict the effect of isoflurane on GLIC. Results from FEP calculations of propofol binding to GLIC are consistent with a model in which propofol blocks the pore at concentrations less than the 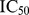, suggesting that the allosteric site observed in X-ray structures [10] may cause a competing potentiation effect.

## Results

### Evolution of the pore region

In control simulations of GLIC without anesthetics, a section of the pore comprising the hydrophobic gate (ILE232–ILE239) tends to constrict slightly over time, which is coupled to breaking of the water column. Spontaneous closure of the pore has also been recently reported in simulations of GLIC with isoflurane, where the pore dehydrated between the gating isoleucines prior to isoflurane binding to the dry ion channel [38]. Although fluctuations in pore width and hydration are to be expected, spontaneous recovery of the open pore conformation was not observed. Systematic closure under simulation suggests that the crystal structure could be more open than typical of the open state, whereas our MD-equilibrated conformations, conversely, seem too constricted to represent an open channel. As a result, it is unclear what conformations are most relevant to the channel's physiological open state. Since large-scale conformational fluctuations cannot be reliably sampled in atomistic simulations, independent simulations were performed to sample both the open-pore and closed-pore conformational basins. Simulations of the closed-pore receptor started from the final configuration of the unrestrained control simulation, where the pore is dehydrated. To simulate the open pore, a set of restraints was designed to prevent closure while minimizing sampling bias within the open-pore basin (see Methods for details).

### Dynamics of bound anesthetic

**Figure 1:**
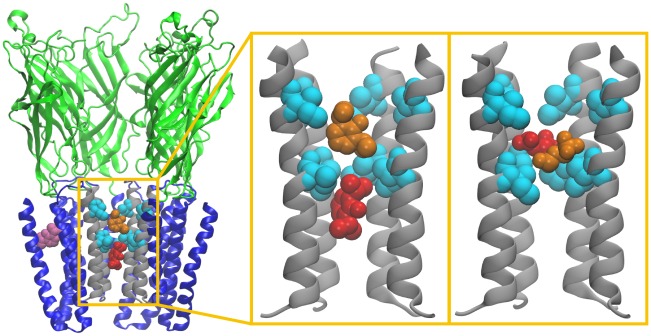
Anesthetic binding sites under investigation. Left: View of the GLIC channel with two propofol molecules blocking a pore restrained to be open (shown in red and orange), and one bound in the crystallographic binding site (purple). Center: The two propofol molecules bound to the pore formed by M2 helices (gray). Right: Analogous magnification of two isoflurane molecules in the pore. Isoleucines bounding the hydrophobic gate (I232 and I239) are shown in cyan. To reveal the pore interior, only four of the five GLIC subunits are shown. An analogous image for the closed conformation is shown in Supporting Figure S1.

**Figure 2:**
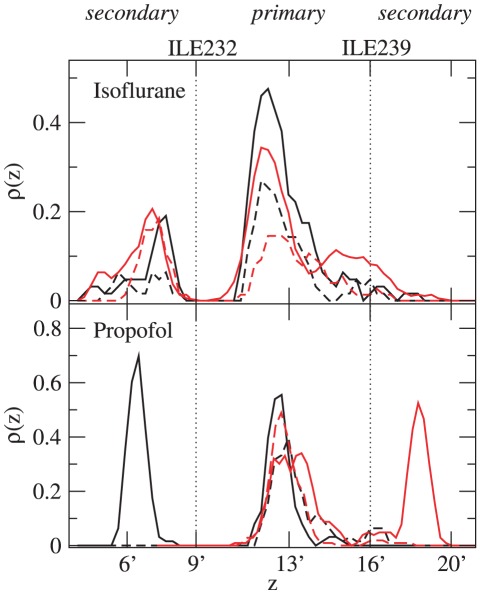
Density distribution of anesthetic molecules in the GLIC pore. Density for one anesthetic bound (dashed) and two anesthetics bound (solid), defined by 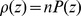, where 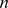 is the number of anesthetics in the pore and 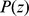 is the probability of observing the anesthetic center of mass in the bin centered around 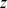. Curves have been smoothed by a three-bin-wide running average. Red: data for the closed pore, black: data for the open pore.

Distributions of the anesthetic molecules' positions along the pore axis (Figures 1–2) indicate a primary binding site between ILE232 and ILE239 (9′ and 16′) for both isoflurane and propofol. This location within the pore corresponds to that observed in crystal structures for the bromine atom of bromo-lidocaine [31] as well the hydrophobic tails of crystallization detergents [27] (Supporting Figure S3). A secondary site is observed between 6′ and 9′ for both isoflurane and propofol, with another secondary site observed between 16′ and 20′ for propofol bound to the closed-pore conformation.

The distribution peaks are substantially wider for isoflurane than for propofol, indicating a looser fit for both primary and secondary sites that is consistent with the relative sizes of the two molecules. Timescales for exchange and hopping among sites are also decreased for isoflurane: exchange between sites was observed in the isoflurane simulations but not the propofol simulations. Figure 2 indicates that secondary sites for propofol had either an occupancy of about 1 or 0 over the course of these simulations, which is consistent with a relatively simple model in which propofol can bind to 2–3 distinct binding sites in the pore.

The same picture cannot be applied to isoflurane. A single isoflurane bound to the closed pore conformation spends similar amounts of time in the primary and secondary site with fractional occupancy (less than 1) in each. The addition of a second isoflurane increases density in the primary site, with occupancy greater than 1 in the primary site and slight increases in occupancy of the secondary site. Therefore, two isoflurane molecules will spend a large fraction of time with the appearance of a dimer, with both molecules in the same local free energy minima, and a small fraction of time in separate minima. This results holds for both pore conformations, although in the closed-pore conformation the dimer is more frequently oriented along the z-axis, as evident by the shoulder on the primary peak; the open-pore primary site has sufficient radius for two isoflurane molecules to occupy similar positions along the z-axis (see, e.g. Figure 1).

During a similar 100 ns traditional simulation of ethanol initially bound to the pore, ethanol exited and re-entered the pore several times, consistent with the low affinities measured by FEP calculations and the absence of effect in electrophysiological studies.

### Predicted binding affinities

Calculated values for 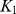 and 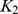 are shown in Table 1, with contributions from various terms shown in Table 2. Propofol has a substantially stronger affinity than isoflurane for all binding sites probed (pore single occupancy, pore double occupancy, and allosteric site). The affinity of ethanol (pore single occupancy) was the weakest measured and indicates negligible binding of ethanol to the pore. The ranking of pore affinities is therefore Propofol
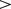
Isoflurane
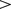
Ethanol, which is the same hierarchy as the GLIC 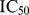, solubility in olive oil, and minimum alveolar concentration (MAC) of these compounds. For propofol and isoflurane, the trend holds for both pore and allosteric sites, suggesting a limited role for such trends in distinguishing among potential mechanisms.

**Table 1:** Predicted binding affinities and experimental 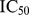.

		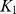	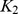	Predicted 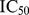 *	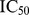
Anesthetic	Binding Site	( 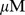 )	( 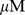 )	( 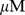 )	( 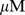 )
Isoflurane	Pore(closed)	2.9	83	2.8–86	60
Isoflurane	Pore(open)	620	530	370–890	60
Isoflurane	Allosteric	2,800			
Propofol	Pore(closed)	0.46	1.8	0.38–2.1	24
Propofol	Pore(open)	1.5	2.9	1.1–4.0	24
Propofol	Allosteric	9.4			
Ethanol	Pore(closed)	7.1 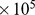			

Binding affinities based on free energies presented in Table 2.

*: Range of predicted 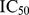 corresponds to model parameter 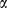 varying from 0 (higher 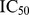) to 1 (lower 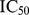).

**Table 2:** Detail of calculated free energies of binding anesthetics to GLIC.

Anesthetic	Binding Site	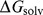	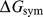	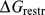	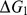	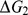
Isoflurane	Pore(closed)	−0.2	0	−0.3	−7.6 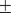 0.2	−5.6 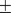 0.4
Isoflurane	Pore(open)	−0.2	0	−0.3	−4.4 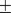 0.9	−4.5 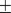 0.7
Isoflurane	Allosteric	−0.2	0	−0.7	−3.5 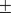 0.3	–
Propofol	Pore(closed)	−1.5	0.4	−0.3	−8.7 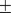 0.2	−7.9 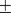 0.9
Propofol	Pore(open)	−1.5	0.4	−0.3	−8.0 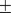 0.6	−7.6 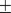 1.2
Propofol	Allosteric	−1.5	0.4	−0.7	−6.9 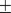 0.2	–
Ethanol	Pore(closed)	−4.6	0.4	−0.3	−0.2 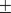 0.3	–

Absolute binding free energies calculated according to Equations 2 and Equations 5. All data expressed in kcal/mol. Error bars correspond to the standard error based on the difference between the recoupling and decoupling calculations.

The affinity of isoflurane for the allosteric site (2.8 mM) suggests relatively weak binding. Broad dose-response curves for GLIC exposed to isoflurane [29], however, indicate that saturation requires concentrations of at least 1 mM; therefore binding of isoflurane to the allosteric site may still contribute to dose response. Measured affinities of isoflurane for the pore sites (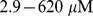, depending on occupancy number and conformation of the pore) are substantially stronger, indicating that at 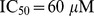 pore-block is a very likely mechanism of inhibition of GLIC by isoflurane.

Measured affinity of propofol for the allosteric site (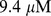) suggests that the allosteric site observed in X-ray structures [10] will likely be occupied at 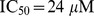. However, like isoflurane, affinity of propofol for the pore sites (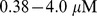) is substantially larger than affinity for the allosteric site. This result is not inconsistent with crystallographic data given that binding of detergents to the pore during crystallization would have prevented observation of pore block by propofol in X-ray structures.

The pore is expected to undergo fluctuations under physiological conditions. The open-pore and closed-pore conformations represent the extremes of this conformational range. Lacking more precise information on the most likely conformations, we calculate affinities for both of these conformations to obtain ranges of possible values. Binding of isoflurane to the pore is strongly conformation-dependent: both affinities measured for the closed-pore conformation are substantially stronger than both affinities measured for the open-pore conformation. As detailed in the following section, this limits precision in predictions of experimental 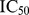 for isoflurane based on a pore-block model alone. The mechanism of propofol binding to the pore appears to be at most weakly conformation-dependent, with affinities for the open pore within error bars of affinities for the closed pore.

Table 1 indicates that binding of isoflurane to the pore is negatively cooperative for the closed-pore conformation (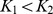) and non-cooperative for the open-pore conformation (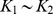). Density of the second isoflurane molecule is found mostly in the primary site, so these results imply that binding of one isoflurane molecule to the whole pore affects the binding of additional molecules to the primary site more strongly in the closed pore than the open pore; this result correlates with the reduced volume available to a dimer in the closed pore relative to the open pore.

Affinities of propofol for the pore indicate a binding mechanism that is at most weakly negatively cooperative. Binding to the secondary site *when the primary site is already occupied* is therefore nearly as favorable as binding to the primary site of an empty pore. This may not be the case when the primary site is unoccupied; however, as previously discussed, sampling limitations preclude us from drawing conclusions about relative affinities for the primary and secondary sites for propofol from Figure 2.

GLIC is not sensitive to ethanol at concentrations ranging up to 200 mM [29], making it a convenient negative control. A single molecule of ethanol was placed in the GLIC pore, and its affinity was calculated as 510 mM, which is consistent with the absence of an effect at lower concentrations. Furthermore, in 100 ns standard MD simulations (non-FEP), the ethanol molecule left the pore, which was not observed in simulations involving either isoflurane or propofol. The results of this negative control suggest that our computational approach for determining strong binding is selective for true high-affinity ligands.

### Prediction of 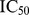 from microscopic parameters

Relating these microscopic parameters to macroscopic properties of the dose-response curve such as the half-maximal concentration (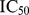) required development of a modified model for dose response due to pore block.

The present model accounts for the possibility that a single anesthetic molecule might not be bulky enough to cause a full block, especially given the observed mobility of smaller anesthetics in the pore. The fractional inhibition 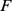 due to pore block by a monomer or dimer can then be described by a modified Adair equation:
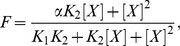
(1)where [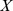] is the concentration of anesthetic and 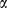 is the probability of pore block by a monomer (it is assumed that the probability of pore block by a dimer is 1). Furthermore, the dissociation constants for binding of the first ligand and the second ligand, are, respectively:
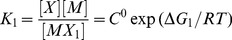
(2)

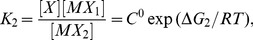
(3)where 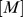 is the concentration of receptors with no anesthetic bound, 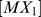 is the concentration of receptors with 1 anesthetic bound, and 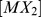 is the concentration of receptors with 2 anesthetics bound, and 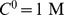. The difference between the present and other more traditional treatments lies primarily in the use of 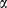 to capture the unknown relationship between occupancy and function.

For 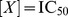, 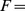 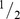, so
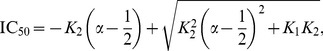
(4)simplifying to 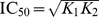 in the special case 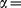 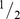. Using 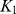 and 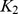 measured from FEP calculations and the upper and lower bounds on physical values of 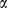 (0 to 1), we predict an 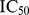 range for isoflurane and propofol (Table 1).

Based on a pore-block model alone, predicted 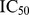 for isoflurane lies in the range 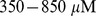 for the open-pore conformation and 2.6–81 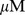 for the closed-pore conformation. The experimental value of 60 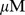, therefore, falls within the range of 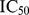 values predicted for these two conformational extremes, although a precise prediction is not feasible due to the uncertainty in the physiological conformation. Only block of functionally open channels is relevant for current inhibition, but the indirect correlation between these extreme structural states and various functional states precludes the simple discounting of the closed-pore conformation. The results do indicate that if the relevant physiological state is more constricted, the 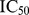 is likely to be larger than if it is more open.

An additional allosteric binding site for the general anesthetics desflurane and propofol in GLIC has been identified using X-ray crystallography [10], although no such site was identified for isoflurane. According to FEP calculations, the affinity of isoflurane for this allosteric site is 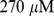, which is weaker than either pore site in either conformation. Dose-response data indicates that saturation, however, requires isoflurane concentrations greater than 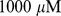, suggesting that the site identified in Ref. [10] will still contribute towards modulation at high concentrations.

Predicted 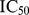 for propofol ranges from 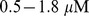 for the open-pore conformation and from 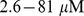 for the closed pore conformation. The pore sites are therefore predicted to be of high affinity, and will likely be occupied at most experimentally accessed concentrations. However, the experimental 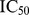 for propofol is 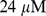 [10] and lies outside the predicted range for all 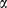, for both closed-pore and open-pore conformations, indicating that a pore-block model alone overestimates the inhibitory effects of propofol. An additional allosteric propofol binding site has been observed in the center of the helical bundles via X-ray crystallography [10].

## Discussion

We have measured microscopic parameters of a potential pore-block mechanism by which inhaled and injected anesthetics inhibit the prokaryotic pentameric ion channel GLIC.

Two sets of simulation conditions were applied: unrestrained conditions under which the transmembrane pore is slightly constricted and hence dehydrated, and restrained conditions preventing such closure and keeping the pore opening similar to that of the crystal structures. To date, there are no reports of unrestrained simulations of GLIC modeled using the CHARMM forcefield in which the pore maintains its crystallographic shape for longer than 25 ns. Possible explanations include limitations of the molecular model, particularly protonation of titratable residues involved in GLIC opening at low pH. Simulation models that allow for fluctuating protonation states are still cumbersome, particularly when many titratable residues are involved.Recently, GLIC was simulated for 200 ns without observed pore collapse [39], using the Amber03 [40] forcefield and the Gromacs software package [41] (The relevant differences between the two approaches have not yet been not been conclusively determined. Performance of CHARMM22/CMAP in tests to detect a native state in an ensemble of potential protein structures is comparable to that of AMBER03 [40].

The extent of the instability is therefore surprising, and may also stem from the presence of six molecules of the detergent DDM in the hydrophobic segment of the pore in at least one crystal (3EAM [27], resolution 2.9 Å); these detergent molecules are not included in most simulations. While other crystal structures for GLIC (including 3EHZ [28], resolution 3.1 Å) do not include resolved detergents in the pore, this may reflect the limited resolution of those structures. As a control, structures were determined by Bocquet et al. [27] from crystals in which DDM is replaced with bulkier brominated analogues. The results indicate that pore occupancy by detergent is reduced, not eliminated, and bound detergent is more disordered. Therefore, the pore conformation resolved crystallographically has not been shown to be independent of occupancy by detergent; this could contribute to the tendency of the empty pore to close in simulations.

A consequence of the likely pore occupancy by crystallization detergent is that little insight into a pore block mechanism can be provided by crystallographic means. Unlike bromo-lidocaine, which has a binding site in the pore [31] that has only partial overlap with that observed for DDM, general anesthetics are predicted to bind to the hydrophobic outer half of the transmembrane pore. The main site, between 9′ and 16′, is fully obstructed by the bundle of DDM molecules (Supporting Figure S3) resolved by Bocquet et al. [27] Therefore, current crystallization conditions for GLIC seem not to be compatible with general anesthetic binding as discussed here.

Both isoflurane and propofol are predicted to bind to the pore as dimers; higher-order aggregates were not investigated since they were not observed in isoflurane “flooding” simulations [24]. Microscopic affinities of one and two anesthetic molecules are calculated using the explicit, formally exact Alchemical Free Energy Perturbation method, and a new analytical model is developed to relate the microscopic affinities to the dose response, including the 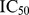 value. The model includes the free parameter 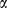, which describes the probability that a single anesthetic will block the pore.

Calculated affinities for isoflurane are highly dependent on pore conformation (Table 1), yielding predicted values of 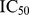 spanning a 300-fold range (2.9 to 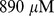), while the experimental value of 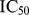 is 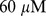. The best agreement with experiment is obtained by considering affinity values for the closed conformation, and assuming that two isoflurane molecules are required to block the pore (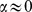). The predicted millimolar affinity of isoflurane for the allosteric site is consistent with the absence of crystal structures resolving isoflurane in the allosteric site. The results, while quantitatively ambiguous due to the uncertainty surrounding details of the GLIC pore structure, are compatible with inhibition of GLIC by isoflurane through pore block.

Propofol is also shown to bind with high affinity to the pore, but a model based on pore block alone overestimates the inhibitory effects of propofol, predicting an 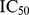 that is at least an order of magnitude lower (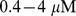) than that measured experimentally (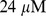). Furthermore, affinity of propofol for the allosteric site observed in crystal structures is 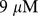, suggesting that both pore and allosteric sites will be occupied over a range of concentrations. In this low concentration domain, a population of receptors will have an allosteric site occupied without block of the pore. We hypothesize that these receptors may actually be potentiated, reducing the observed net inhibition caused by pore block and thereby increasing the apparent 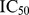. Such competing effects may explain why propofol dose response is not well described by a Monod-Wyman-Changeux model.

This proposed model is consistent with a current model for competing effects in the nicotinic Acetylcholine Receptor and at super-clinical anesthetic concentrations in the 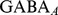 receptor [17]. Due to the possibility that binding to the intrasubunit allosteric site could cause either gain or loss of function, interpreting mutagenesis results such as those reported with the crystal structure in Ref. [10] becomes challenging: observed reduction in the 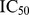 could result from a mutation that causes a higher affinity for a negative modulator or lower affinity for a positive modulator. Given such ambiguities, photoaffinity labeling of GLIC with azi-propofol [42] or azi-isoflurane [43] in the presence and absence of known GLIC channel blockers [44], [45] may provide the most straightforward experimental test of a pore-block mechanism. This work highlights the subtleties involved in interpreting global functional measurements when competing binding sites are likely.

The ranking of pore affinities corresponding to rankings in GLIC 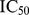, solubility in olive oil, and minimum alveolar concentration (MAC) of these compounds, with propofol
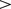
isoflurane
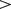
ethanol. Most known anesthetics follow the classic Meyer-Overton [1], [2] correlation that linearly relates MAC to solubility in olive oil; this correspondence can be extended to exclude non-immobilizers when lipophilicity is substituted by affinity for the hydrophobic-polar interface [46]. The GLIC pore contains both hydrophobic and polar residues, forming an interfacial environment which will likely bind a wide range of amphipathic general anesthetics, as well as charged local anesthetics and other blockers [44], [45]. The 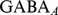 receptor transmembrane pore is noticeably less hydrophobic at the location where we predict anesthetic binding: this could result in a lower affinity for anesthetic and the lack of clinically significant pore block in the 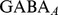 receptor: such a difference would be crucial in explaining the opposing effects of general anesthetics on inhibitory and excitatory receptors.

Use of conformational restraints on the pore in some of the present affinity calculations aims at probing two end-points within a conformational ensemble, in the absence of reliable knowledge of the actual conformational distribution forming the open state of GLIC. This approach is vindicated by the propofol results, which prove insensitive to the restraints, or lack thereof. Therefore, the predicted propofol affinities are the main, unambiguous basis to our hypothesis of counteracting effects between sites. In contrast, isoflurane binding is more sensitive to the pore conformation, and the experimental 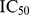 falls between those predicted from the two conformational extremes. Thus the isoflurane affinities for the pore are obtained with a significant margin of uncertainty, and should be regarded merely as an argument for continued plausibility of pore block as a potential mechanism for modulation by isoflurane.

Although historically, pore block has not been widely discussed as a potential molecular mechanism for the action of general anesthetics, periodic results from electrophysiology [32], mutagenesis [11], photoaffinity labeling [17], and simulation [24], [26] have suggested the pore as a site for inhibition by anesthetics in eukaryotic Cys-loop receptors. The simulations in the present work offer quantitative results regarding the affinities of general anesthetics for the pore of a ligand-gated ion channel; the robustness of this finding lead us to propose pore block as a significant mechanism for channel inhibition by general anesthetics.

## Methods

### System setup

Initial GLIC coordinates were taken from the 3EAM structure, and prepared in a similar manner to previous studies of GLIC [24], [27]. GLIC was protonated according to the Henderson-Hasselbalch relationship with the pKa results given in Ref. [27], and protonation states on ionizable residues assigned assuming a pH of 4.6. The protein was placed in a pre-equilibrated 1-palmitoyl-2-oleoyl-sn-glycerol-phosphatidylcholine bilayer that was originally created with the MEMBRANE plugin of VMD [47]. Following protein insertion, the individual anesthetics were placed into either the pore or the site determined by X-ray crystal structure. To place anesthetics in the channel itself, the center of mass (COM) of the anesthetic was moved with a random orientation to overlap with the COM of the pore. Here, we identify the COM of the pore as the COM of the C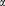 atoms of residues Tyr226 and Tyr244. For the anesthetics placed in the X-ray site, the COM of individual anesthetics were moved to overlap with the COM of the C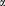 atoms defined as the boundary of the binding site from the residues on M1, M3, M4, and the 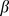
6–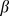
7 loop given in Ref [10]. After the anesthetics were placed in the binding site, the system was solvated using the SOLVATE command in VMD, and NaCl was added to bring the salt concentration to 0.15 M. In the end, system was comprised of roughly 200,000 atoms including water, protein, membrane, ions, and anesthetics.

### Simulation details

Ten thousand steps of minimization were run to remove any bad initial contacts, followed by 2 ns of equilibration in which both protein and anesthetic were harmonically restrained with a 5 kcal/mol/Å force constant to their initial positions. This allowed the membrane and aqueous solution to relax around the protein-anesthetic complex. A second 2 ns simulation was run where only the anesthetic was positionally restrained, yet all other atoms including those in the protein, were relaxed around it. Following this equilibration process, a simulation was run for between 50–100 ns for each of the eight systems shown in Figure 2, with flat-bottom pore restraints (described below) on the “open-pore” simulations. In total, including a 300 ns control simulation with no anesthetics, the traditional MD systems required 1.2 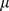
s of aggregate simulation time, and the ending coordinates of these simulations were used as input to their corresponding FEP calculations.

All simulations used the CHARMM22-CMAP force field with torsional corrections for proteins [48], [49]. The CHARMM27 [50] model was used for phospholipids, ions, and water, ethanol parameters were taken from the CHARMM35 model [51], while parameters previously developed in our group were used for isoflurane. New CHARMM-consistent force field parameters for propofol were developed for this study. All parameters can be found in Supporting Dataset S1 and Supporting Dataset S2 and a description of the parameterization process can be found in Supporting Text S1. Energy minimization and MD simulations were made by the NAMD2.7b2 package [52]. All simulations employed periodic boundary conditions, long-ranged electrostatics were handled with the smooth particle mesh Ewald method [53], and a cutoff of 1.2 nm was used for Lennard-Jones potentials with a smoothing function applied starting at 1.0 nm. All simulations were run in the NPT emsemble with weak coupling to a Langevin thermostat and barostat at a respective 300 K and 1 atm. All bonds to hydrogen atoms were constrained using the SHAKE/RATTLE algorithm. A multiple timestep rRESPA method was used, and controlled with a high-frequency timestep of 2 fs and a low-frequency timestep of 4 fs. RMSD plots are shown in Supporting Figures S4 and S5.

### Pore restraints

Closing of the pore was found to be well-described by pairwise distances between M2 helices across the pentamer. For each M2 helix, the geometric center of alpha carbon atoms in the extracellular half (residues ILE232 to LEU245) was determined. Five distances 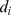 between these centers were calculated, between all pairs of non-adjacent subunits: 1-3, 1-4, 2-4, 2-5, 3-2. Statistical distributions of these distances from an unrestrained simulation show two peaks: a smaller one corresponding to the metastable open basin around 20 Å, and a larger population in the collapsed state, at distances smaller than 19 Å. From the distributions, free energy profiles may be extracted: although incomplete because of insufficient sampling, they do give an indication about the shape of the metastable open basin and the barrier separating it from the collapsed state. (Supporting Figure S2) Based on this information, we designed a “flat-bottom” restraint potential which enhances the effect of the barrier and traps the pore in its open conformation, without altering the energy landscape within the open basin. The potential is zero for 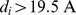, and a harmonic wall with a force constant of 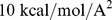 at smaller values of 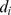 (Supporting Figure S2). Restraints were applied through the Collective Variables module of NAMD [54].

### FEP calculations

The total microscopic free energies of binding are
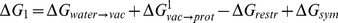
(5)

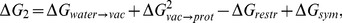
(6)where 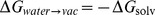, 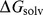 represents the solvation free energy of the anesthetic, 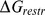 represents the entropic cost of imposing the restraints relative to the standard state volume, 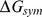 is the penalty due to loss of symmetry upon binding [55], 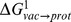 is the free energy required to move a single anesthetic from vacuum into an unoccupied binding site, and 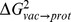 is the free energy required to move a single anesthetic from vacuum into a binding site already occupied by one anesthetic. 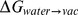 varies for each anesthetic and was calculated using the protocol in Ref. [37]. 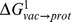 and 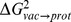 require, by far, the most calculation time. These values are measured, as in Ref. [37], by successively decoupling and recoupling interactions between the anesthetic and the protein/water/ion/other anesthetic environment, over a series of windows. For the systems with anesthetics in the pore, flat-bottom cylindrical harmonic restraints were applied, while flat-bottom spherical restraints were applied to keep anesthetics in the X-ray binding site. For measurements of 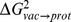 , only one anesthetic in the pore underwent recoupling/decoupling, while both were subject to a cylindrical restraint. The force constants for both binding site restraints were on the order of 10 kcal/mol/Å, and implemented in similar fashion to methods used in our previous simulations of the binding of isoflurane to apoferritin [37]. Due to the “square-well” shape of the restraint, 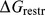 is given purely by the entropic cost of constraining the anesthetic within the bounds of the restraint relative to a standard state solution:
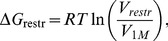
(7)where 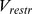 is the volume enclosed by the restraint and 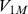 is the free volume the ligand would have in the standard state (a 1 M solution). For calculations in the pore site, 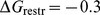 kcal/mol, while for calculations in the allosteric site, 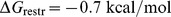. 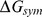 for binding of A and B is equal to 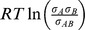 where 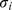 is the symmetry number of species 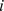 [55], in our case 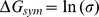, where 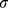 is one for isoflurane, and two for propofol or ethanol. For the latter, the symmetry term contributes 0.4 kcal/mol to binding free energies or a factor of two in binding affinity.

In the present work the perturbation parameter 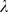 was sampled with a step size equal to 0.025 between 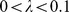 and 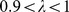, and 0.05 otherwise. Each step in 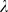 started with a 4 ps equilibration period followed by a 1 ns run for data collection, and each decoupling and recoupling stage required 25 ns of simulation. To avoid the so-called “end point catastrophy”, a soft-core potential of Zacharias [35], [36], [56] was used, both electrostatics were decoupled from each other during the FEP runs, and Lennard-Jones potentials were shifted by 6.0 Å. Each system was decoupled and recoupled once, requiring a total of 50 ns of FEP simulations for each of the eleven systems, totaling to 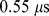 of FEP data overall. Free energies were estimated by combining the three decoupling runs into one data set, the three recoupling runs into one data set, and then combining backwards and forwards runs using the Bennett acceptance ratio method [57]. However, the use of the Bennett analysis method did not affect the results significantly, relative to a simple averaging of free energies measured from individual recoupling and decoupling stages.

## Supporting Information

Dataset S1 **Propofol parameter file in CHARMM format, including CMAP terms.**
(TXT)

Dataset S2 **Propofol topology file in CHARMM format.**
(TXT)

Figure S1 **Anesthetic binding sites in the unrestrained pore conformation.** Left: View of the GLIC channel with two propofol molecules blocking an unrestrained pore (shown in red and orange), and one bound in the crystallographic binding site (purple). Center: Two propofol molecules in the pore formed by M2 helices (gray). Right: Analogous magnification of two isoflurane molecules in the pore. Isoleucines bounding the hydrophobic gate (I232 and I239) are shown in cyan. To reveal the pore interior, only four of the five GLIC subunits are shown.
(TIF)

Figure S2 **Design of the collective variable restraints.** Solid lines: Boltzmann transform of the distribution of the five distances between M2 helices (see Methods) in an unbiased control simulation. Dotted line: flat-bottom restraint potential applied to confine the pore to the open basin.
(EPS)

Figure S3 **Location of crystallographic DDM detergent and bromo-lidocaine in the GLIC pore.** M2 helices of GLIC are shown as grey cylinders (one omitted for clarity), with isoleucine residues 232 and 239 as cyan spacefill. DDM molecules from structure 3EAM [27] are shown as sticks (one omitted for clarity). Bromine atom of bromo-lidocaine from structure 2XQ3 [31] is shown as an orange sphere.
(TIF)

Figure S4
**Root mean square deviations (RMSD) averaged over all C** 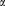 **atoms in the protein.** Black lines represent the open pore, red lines represent the closed pore, solid lines are for the doubly occupied pore, and dashed lines are for the singly occupied pore. For the closed pore occupied by a single isoflurane, two trajectories have been used. In this case, one trajectory is given as a dotted line and one by a dashed line.(EPS)

Figure S5
**Root mean square deviations (RMSD) averaged over C** 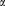 **atoms in the M2 helices.** Black lines represent the open pore, red lines represent the closed pore, solid lines are for the doubly occupied pore, and dashed lines are for the singly occupied pore. For the closed pore occupied by a single isoflurane, two trajectories have been used. In this case, one trajectory is given as a dotted line and one by a dashed line.(EPS)

Text S1 **Method for propofol parameterization.**
(PDF)
