# Supplementary figures and images for: General Anesthetics Predicted to Block the GLIC Pore with Micromolar Affinity
Source: PLoS Comput Biol. 2012 May 31;8(5):e1002532. doi: 10.1371/journal.pcbi.1002532 (PMC3364936; doi:10.1371/journal.pcbi.1002532)

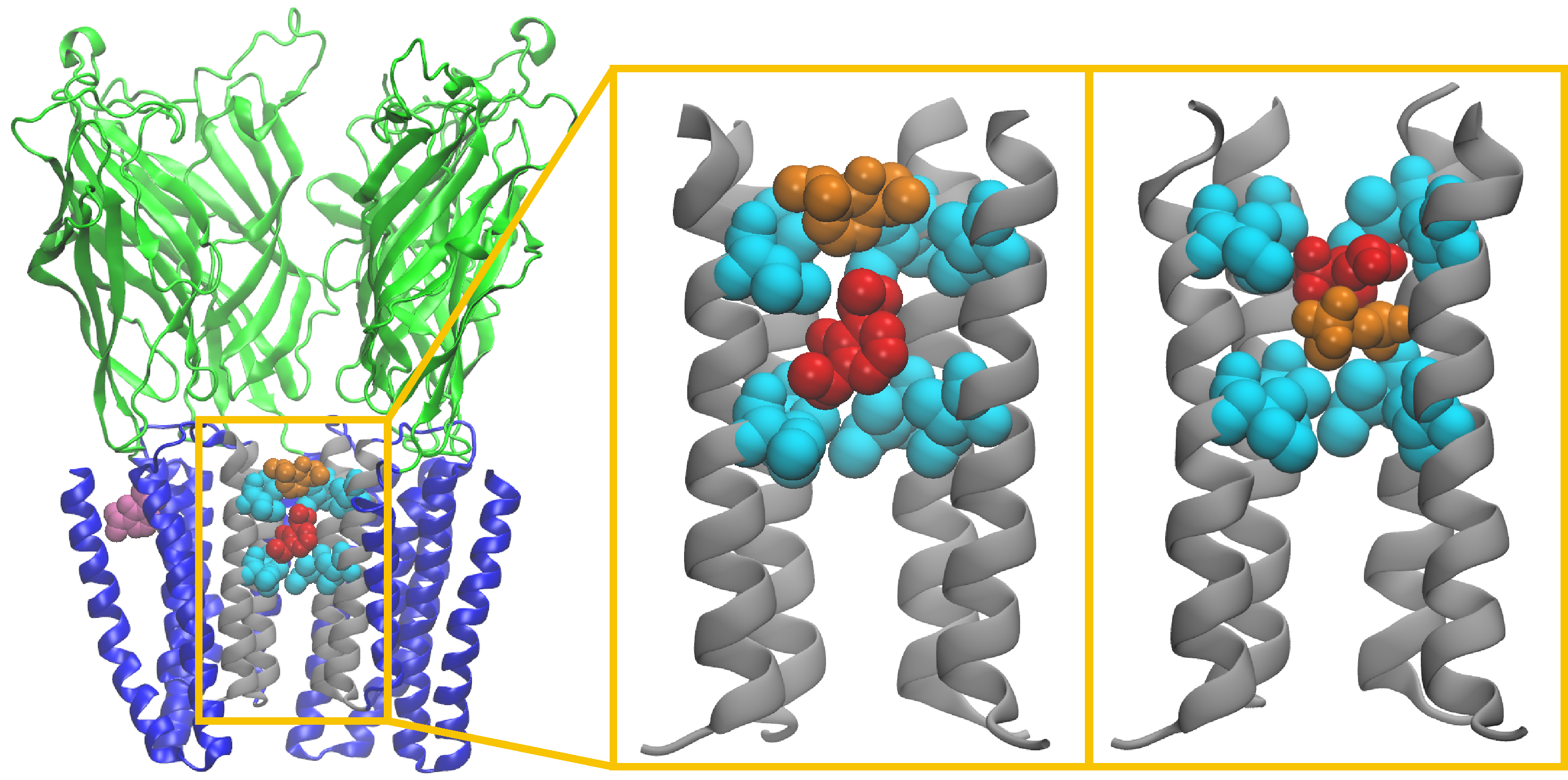

Supplement: Figure S1 — Anesthetic binding sites in the unrestrained pore conformation. Left: View of the GLIC channel with two propofol molecules blocking an unrestrained pore (shown in red and orange), and one bound in the crystallographic binding site (purple). Center: Two propofol molecules in the pore formed by M2 helices (gray). Right: Analogous magnification of two isoflurane molecules in the pore. Isoleucines bounding the hydrophobic gate (I232 and I239) are shown in cyan. To reveal the pore interior, only four of the five GLIC subunits are shown. (TIF) [file pcbi.1002532.s003.tif]

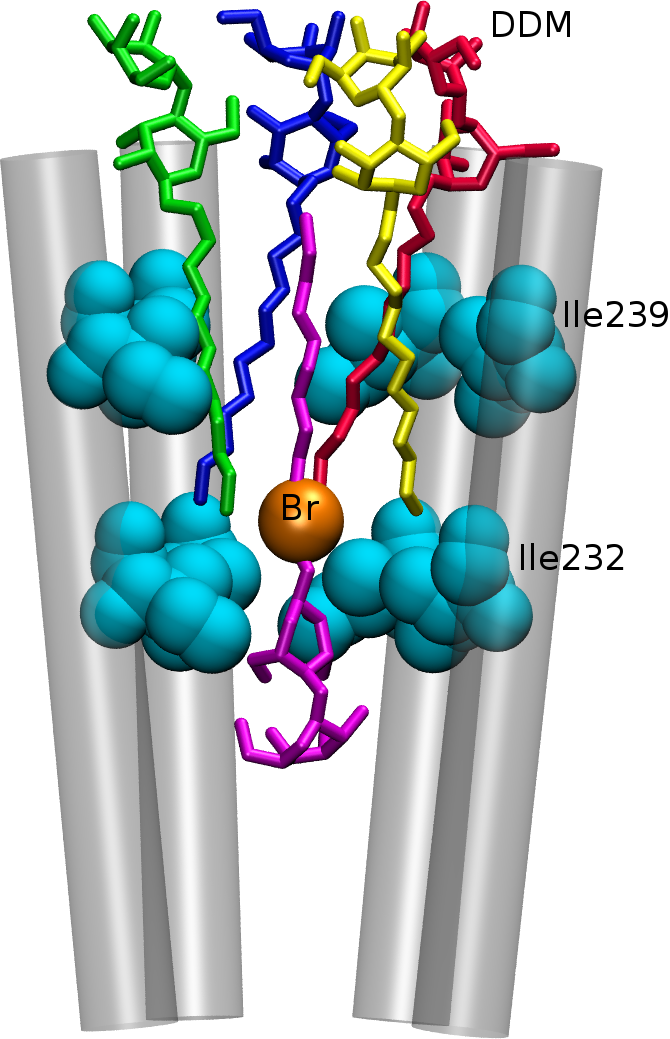

Supplement: Figure S3 — Location of crystallographic DDM detergent and bromo-lidocaine in the GLIC pore. M2 helices of GLIC are shown as grey cylinders (one omitted for clarity), with isoleucine residues 232 and 239 as cyan spacefill. DDM molecules from structure 3EAM [27] are shown as sticks (one omitted for clarity). Bromine atom of bromo-lidocaine from structure 2XQ3 [31] is shown as an orange sphere. (TIF) [file pcbi.1002532.s005.tif]
