# Supplementary material for: General Anesthetics Predicted to Block the GLIC Pore with Micromolar Affinity
Source: PLoS Comput Biol. 2012 May 31;8(5):e1002532. doi: 10.1371/journal.pcbi.1002532 (PMC3364936; doi:10.1371/journal.pcbi.1002532)
Supplement: Text S1 — Method for propofol parameterization. (PDF) [file pcbi.1002532.s008.pdf]

Text S1 : Parameterization of propofol

David N. LeBard, Jérôme Hénin, Roderic G. Eckenhoff,
Michael L. Klein, Grace Brannigan

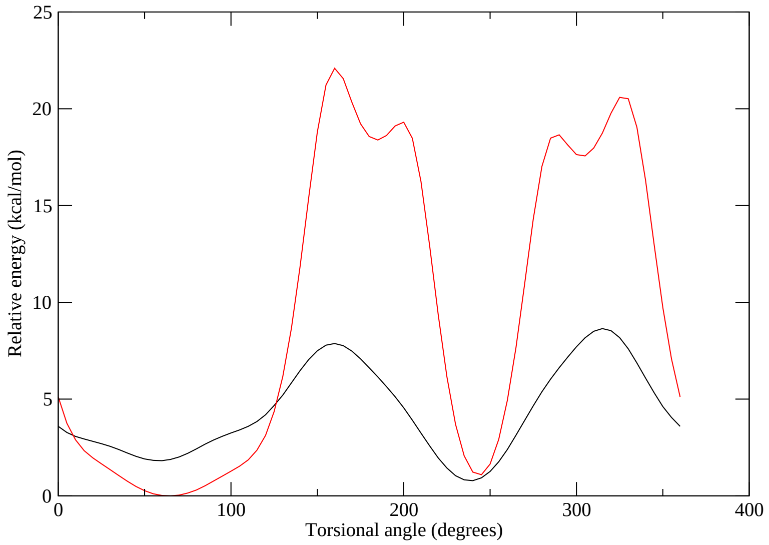

Figure 1: **Ab initio potential energy surfaces for the isopropyl/phenol torsional angle when the hydroxyl is facing away (black) or toward (red) the isopropyl.** Torsional angle corresponds to C11-C7-CE1-CD1 or C10-C8-CE2-CD2 (atoms defined in Dataset S2).

Electronic copies of the CHARMM-formatted parameter and topology files with CMAP torsional corrections for propofol have been included as supporting information (Dataset S1 and Dataset S2, respectively). Propofol parameters were determined by combining pre-existing CHARMM parameters [1] for isopropyl and phenol, leaving only dihedral parameters corresponding to rotation of isopropyl and phenol groups around their adjoining bond to be determined. The potential energy surface for such rotations was calculated using NWChem [2] and a relaxed scan, at the DFT level with the B3LYP [3,4] hybrid exchange-correlation functional and the 6-311++G(2d,p) basis set. Potential energy surfaces for the rotation were determined for the cases in which the phenol hydroxyl faces both toward and away from the isopropyl group (Fig. 1). The results indicate that the minimum energy conformation of this dihedral is dependent upon its proximity to the phenol hydroxyl, yielding an asymmetry in minimum energy conformations for the two isopropyl groups.

This behavior can be modeled using the CMAP parameters [5] of the CHARMM forcefield, which influence correlations among different dihedrals. A relaxed scan

was conducted over both the C11-C7-CE1-CD1 dihedral and the CE2 CZ O1 H2 dihedral to generate the 2D potential energy surface. A classical torsional scan using the CHARMM forcefield in which the C11-C7-CE1-CD1/C10-C8-CE2-CD2 dihedral potential was set to 0 was carried out, and CMAP terms were calculated to correct this classical potential energy surface.

References

[1] MacKerell Jr AD, Bashford D, Bellott M, Dunbrack Jr RL, Evanseck JD, et al. (1998) All-atom empirical potential for molecular modeling and dynamics studies of proteins. J Phys Chem B 102: 3586–3616.

[2] Valiev M, Bylaska E, Govind N, Kowalski K, Straatsma T, et al. (2010) Nwchem: a comprehensive and scalable open-source solution for large scale molecular simulations. Comput Phys Commun 181: 1477.

[3] Lee C, Yang W, Parr R (1988) Development of the Colle-Salvetti correlation-energy formula into a functional of the electron density. Phys Rev B 37: 785–789.

[4] Becke AD (1993) Density-functional thermochemistry. III. The role of exact exchange. J Chem Phys 98: 5648.

[5] MacKerell AD, Feig M, Brooks CL (2004) Improved treatment of the protein backbone in empirical force fields. J Am Chem Soc 126: 698-9.
